# Supplementary material for: Canopy soil bacterial communities altered by severing host tree limbs
Source: PeerJ. 2017 Sep 6;5:e3773. doi: 10.7717/peerj.3773 (PMC5591635; doi:10.7717/peerj.3773)
Supplement: File S1 [file peerj-05-3773-s001.pdf]

Canopy soil bacterial communities altered by severing host tree limbs

Cody R. Dangerfield, Nalini M. Nadkarni, William J. Brazelton

Supplementary File S1
Additional photographs of the study area, experimental treatments, and samples.
Photo credits: N. Nadkarni

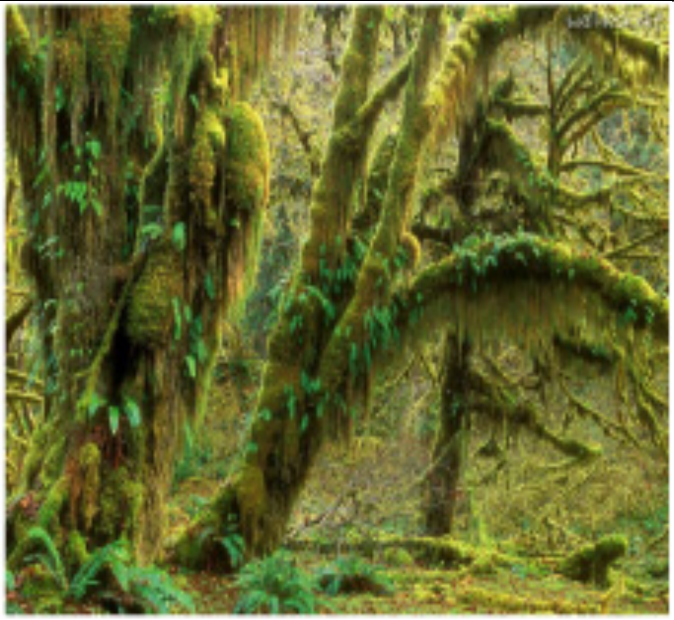

1. Temperate rainforest study site

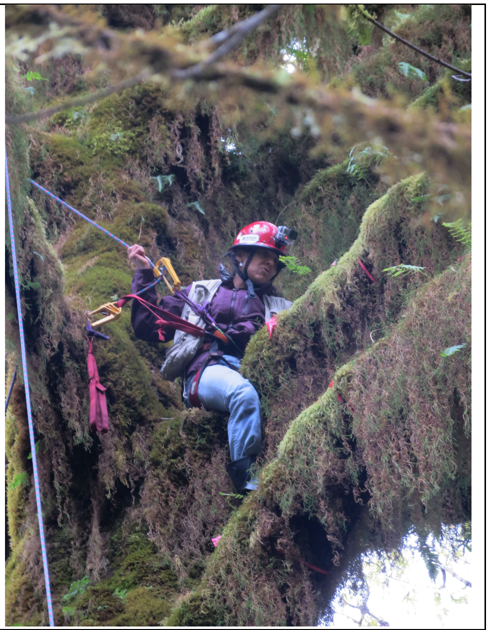

2. Canopy sampling

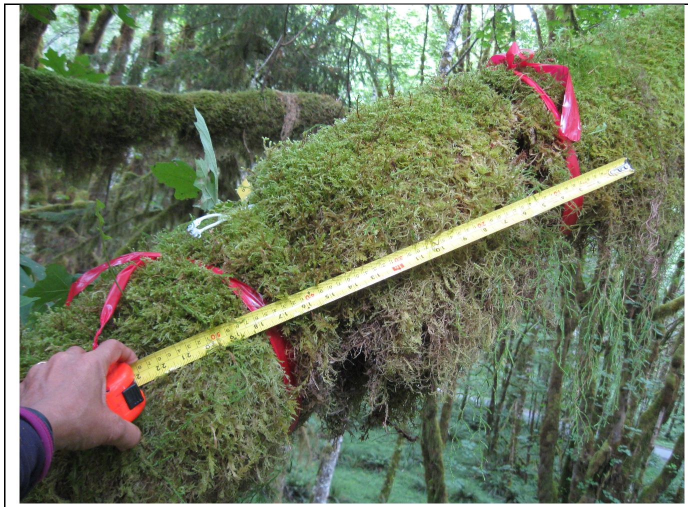

3. Canopy-original

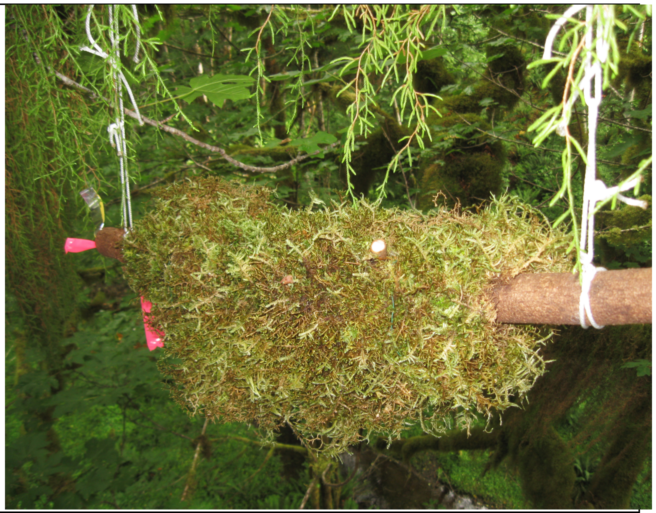

4. Canopy-severed

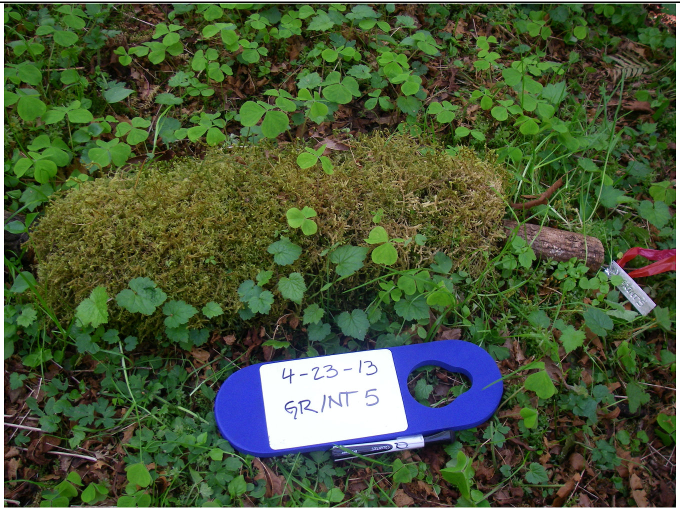

5. Ground-perched

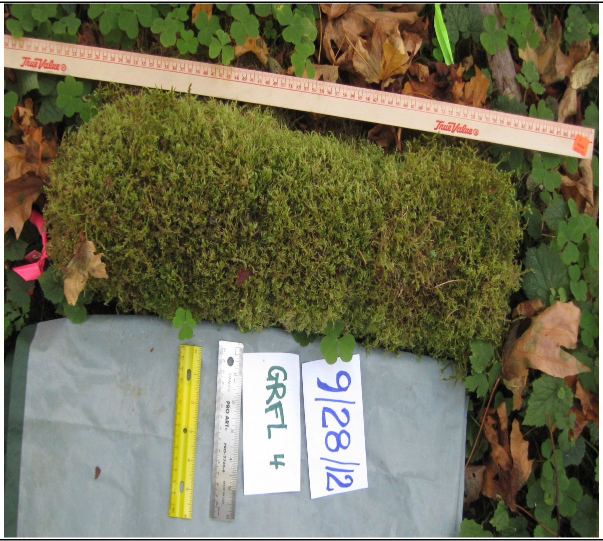

6. Ground-flat

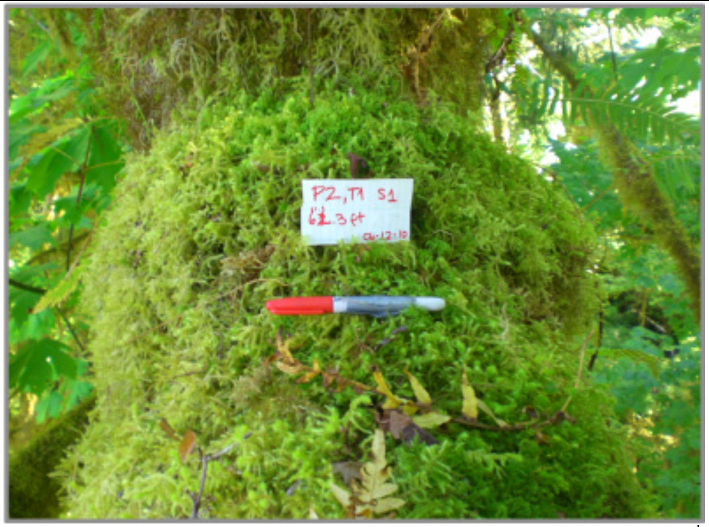

7. Undisturbed epiphytic material

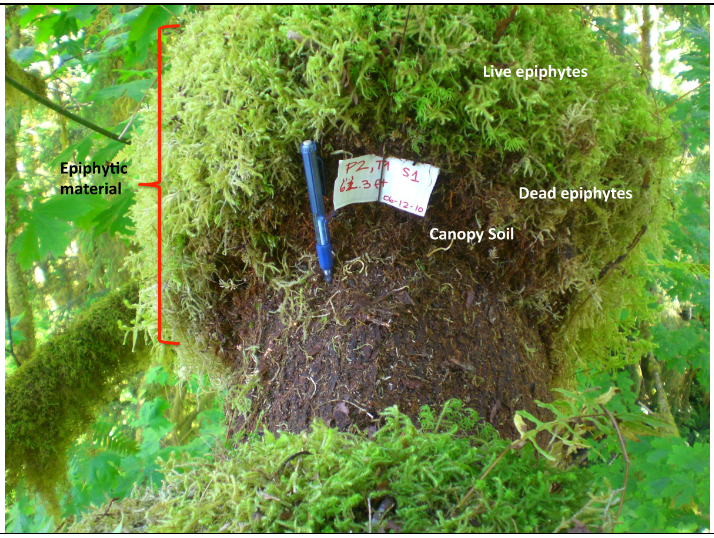

8. Epiphytic material with exposed canopy soil

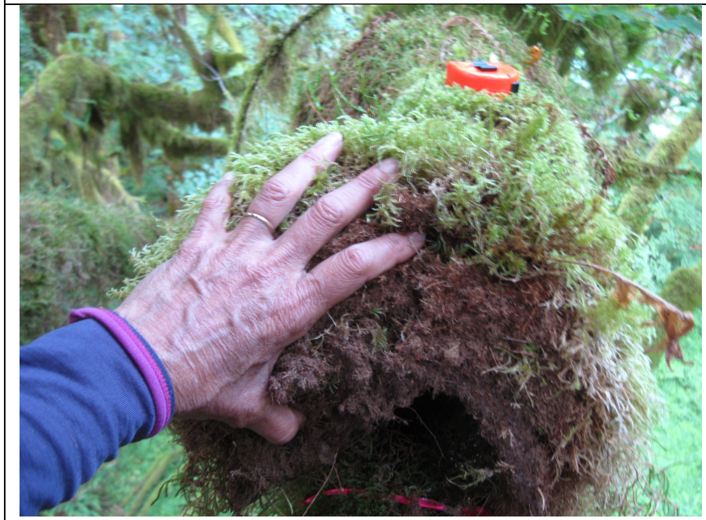

9. Canopy soil profile

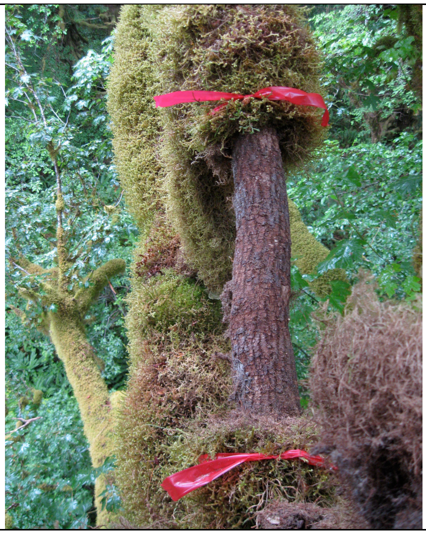

10. Branch with epiphytic material removed
